# Novel TMS-derived metrics enable machine learning classification of major depressive disorder

**DOI:** 10.1038/s44277-025-00053-w

**Published:** 2026-01-12

**Authors:** Santiago López Pereyra, Diego R. Mazzotti, Desmond Oathes, Jennifer R. Goldschmied

**Affiliations:** 1 Department of Mathematics, Astronomy, Physics and Computer Science, National University of Córdoba, Córdoba, Argentina; 2 Division of Medical Informatics, Department of Internal Medicine, University of Kansas Medical Center, Kansas City, KS USA; 3 Department of Psychiatry, University of Pennsylvania, Philadelphia, PA USA

**Keywords:** Learning algorithms, Depression

## Abstract

No validated biomarker currently exists for early detection or personalized treatment of major depressive disorder (MDD). Transcranial magnetic stimulation (TMS) is widely used in clinical and research settings and holds promise for biomarker discovery. We assessed two novel TMS-derived cortical excitability metrics, $\delta$ and $\varrho$, for distinguishing individuals with MDD from healthy controls. Motor-evoked potentials (MEPs) were recorded from the left abductor pollicis brevis during TMS of the right primary motor cortex in twenty-six unmedicated MDD patients and seventeen never-depressed controls. $\delta$ and $\varrho$ were computed from peak-to-peak MEP amplitudes. A Gradient Boosting classifier predicted diagnostic status using raw MEPs, $\delta$ and $\varrho$, or their combination. While MEPs alone were non-predictive, $\delta$ and $\varrho$ significantly improved accuracy. Combining MEPs with $\delta$ and $\varrho$ yielded 83.3% accuracy and 82.3% balanced accuracy. These results suggest $\delta$ and $\varrho$ effectively capture neurophysiological alterations in MDD and support their potential as candidate biomarkers for MDD.

## Lay Summary

Diagnosing major depressive disorder (MDD) is challenging because the condition has a wide array of presenting symptoms. Thus, a combination of multiple biomarkers capturing different aspects of the condition is needed. We formulated two novel proxies of cortical excitability using transcranial magnetic stimulation (TMS). Combined with a simple machine learning model, these metrics predicted depression with >82% accuracy. This fills an important gap, since previously proposed biomarkers were not typically based on cortical excitability.

## Introduction

Major depressive disorder (MDD) is one of the leading causes of poor health and disability worldwide [1]. Despite its pervasiveness, its heterogeneous presentation [2] has made it difficult to identify valid biomarkers that can accurately identify individuals with this condition. This limits early detection or the ability to develop personalized medicine approaches.

Recent research stresses the need to develop a composite set of multiple biomarkers which can potentially outperform individual ones [3]. Some proposed biomarkers of MDD include abnormal functional connectivity and activity in networks important to affective and cognitive processing, such as the anterior cingulate cortex, amygdala, and dorsolateral prefrontal cortex [4–6]; inflammatory cytokines and neuroinflammation, which are consistently elevated in depressed individuals [7, 8]; decreased levels of brain-derived neurotrophic factor (BDNF) [9–11], and dysregulation in the expression of a wide array of non-coding RNAs [12, 13]. Interestingly, even though current theories suggest that MDD involves impairments in synaptic excitability and strength [14–17], no biomarkers based on these factors have yet been proposed.

In this context, proxy measures of cortical excitability derived from transcranial magnetic stimulation (TMS) offer a promising avenue for identifying new candidate biomarkers. TMS is a non-invasive approach used as a treatment for MDD. During TMS, a magnetic field is applied to a predetermined location on the scalp in order to modulate the electrical activity in the corresponding part of the brain. When TMS is applied to the motor cortex, it is possible to measure the motor evoked potential (MEP) via an electromyography (EMG) of the targeted muscle. The MEP is suggested to index the aggregate effect of excitatory and inhibitory potentials in the corticospinal motor pathways, therefore serving as a functional proxy of cortical excitability [18–24]. TMS studies consistently show that corticospinal excitability is altered in patients with MDD [25–33]. By providing a functional index of corticospinal excitability, TMS measures could fill an important gap in the development of a biomarker panel.

The potential of TMS for developing biomarkers is not novel, as it has been suggested in conditions such as epilepsy and various kinds of neurodevelopmental disorders [34–36]. However, methodological limitations make the translation of TMS-evoked MEPs to clinical biomarkers difficult. When proxying excitability with MEPs, their aggregated peak-to-peak amplitude is typically used. But MEPs have a high within subject, between-subject, and across session variability [37–39]. This not only means that aggregation sacrifices potentially informative variability in the data, but also raises concerns about how representative these aggregates are in the first place. Additionally, aggregation significantly downscales data resolution, rendering data driven methods of analysis impossible.

To address this, we designed two novel TMS-derived measures of cortical excitability termed $\delta$ and $\varrho$. These quantify the peak-to-peak amplitude of MEPs relative to the mean (arithmetic or harmonic) peak-to-peak amplitude of some baseline set of stimulations. $\delta$ and $\varrho$ involve no data reduction, avoid the limitations of aggregation-based proxies of cortical excitability, and are easy to interpret in a clinical setting.

Thus, the goal of the present study was to evaluate the utility of $\delta$ and $\varrho$ in predicting MDD status with machine learning (ML) models. We hypothesize that the ML model trained on these metrics will be able to predict MDD status with high accuracy, providing a novel tool for enhancing the diagnosis of MDD and potentially a novel biomarker for clinical use.

## Materials and methods

### Participants

Participants were recruited from the Philadelphia metropolitan area as part of a larger study investigating the effects of slow-wave sleep modulation on cortical excitability. Eligibility criteria required participants to be aged 25 to 50 years, right-handed, and to have a habitual sleep duration of 6–9 h per night, with a bedtime between 9 PM and 12 AM. Exclusion criteria included any history of general medical illnesses, significant head injuries, seizures, or loss of consciousness exceeding five minutes. Participants were also excluded if they were on psychotropic medications or recreational drugs. However, participants on psychotropic medication who wished to join the study were allowed to discontinue their medication under the guidance of a study psychiatrist and could participate after being medication-free for at least two weeks, or four weeks for those on fluoxetine. Psychotropic medications were considered exclusionary as changes to brain neurochemistry not due to experimental procedures would confound study results, reducing rigor. Female participants were neither pregnant nor breastfeeding. Additionally, participants had to be free of sleep disorders, such as narcolepsy, sleep apnea, bruxism, or periodic limb movements, and could not be involved in shift work, as confirmed by medical history or polysomnography. Informed written consent was obtained from all participants, and the study protocol received approval from the Institutional Review Board at the University of Pennsylvania.

The study sample included 26 adults (16 females) diagnosed with MDD according to the Structured Clinical Interview for DSM-V (SCID). All participants fulfilled the criteria for a current major depressive episode. Concurrent anxiety disorders or attention deficit and hyperactivity disorder were allowed, but individuals with a history or current diagnosis of psychosis, obsessive-compulsive disorder, post-traumatic stress disorder, or substance use disorder were excluded. Symptom severity was evaluated using the Hamilton Depression Rating Scale (HAM-D). Additionally, the sample included 17 healthy adults (7 females) who were confirmed through the SCID to have no current or past history of psychopathology.

### Procedures

Prior to the laboratory study, participants adhered to a five-day fixed sleep schedule based on their usual sleep patterns, and refrained from napping. Compliance with this schedule was monitored through sleep diaries and actigraphy. Participants also agreed to abstain from alcohol and limit their caffeine intake to one cup per day before 12:00 PM on the days of the study overnights. Each participant spent two non-consecutive nights in the laboratory, with at least one week and no more than three weeks between visits. The present analysis focuses solely on the baseline night of the experimental procedure. On the morning following each overnight stay, participants underwent the TMS protocol, which included the assessment of resting motor threshold (RMT) and intracortical facilitation/inhibition.

### Transcranial magnetic stimulation (TMS)

During the TMS protocol participants were comfortably seated with their hands at rest. TMS pulses were administered using a MagPro X100 stimulator (MagVenture Inc, Alpharetta, GA) equipped with a figure-of-eight coil. The coil was positioned over the right primary motor cortex (M1), and surface EMG recordings were taken from the abductor pollicis brevis (APB) muscle in the left hand. The EMG data window was set to 15 ms and 515 ms post-stimulation. Any pre-TMS muscle contraction and TMS pulse artifact, identified within a −15 to 15 ms window around the TMS pulse, were manually rejected based on visual inspection (less than 2% of data were excluded). The RMT was defined as the minimum intensity required to elicit a motor evoked potential of 50 μV in at least 5 out of 10 trials from the corresponding EMG and was determined before each test session.

### Intracortical facilitation (ICF) and inhibition (ICI)

ICF and ICI were assessed using a paired-pulse TMS protocol in which two stimuli were delivered with interstimulus intervals (ISIs) of 4, 5, 8, 10, 15, or 20 ms. The first (conditioning) stimulus was set at 80% of the RMT, followed by a second (conditioned) stimulus at 120% of the RMT to evoke a motor-evoked potential. Eight MEPs were recorded for each ISI. The paired pulses were randomly intermixed with 24 single pulses, totaling 72 MEPs in each test session. The intertrial interval was randomly set at either 5 or 6 ms.

### Measures of cortical excitability

TMS-induced MEPs are modulated by a wide array of neurobiological factors and capture corticospinal excitability in stochastic fashion [40]. Thus, we modelled them via random variables, which following the empirical distribution of our samples we assume to be Gamma-distributed.

In particular, let $X \sim \varGamma ({\alpha }_{X},\,{\theta }_{X})$ denote the peak-to-peak amplitude of the MEP evoked by a (single or paired) TMS. Let ${T}_{1},\ldots ,\,{T}_{m}$, with ${T}_{i}\, \sim \,\varGamma ({\alpha }_{T},\,{\theta }_{T})$ for $1\le i\le m$, denote the peak-to-peak amplitude of the MEP evoked by $m$ single, distinct stimulations. In general, we allow that $X={T}_{i}$ for some ${T}_{i}$, in which case ${\alpha }_{X}={\alpha }_{T},\,{\theta }_{X}={\theta }_{T}$. We define our two measures of cortical excitability as follows:

$$\varrho =\frac{{Xm}}{{\sum }_{i=1}^{m}{T}_{i}}$$

$$\delta =\frac{X}{m}{\sum }_{i=1}^{m}\frac{1}{{T}_{i}}$$

Thus, $\delta$ and $\varrho$ correspond to the ratio of a MEP to the harmonic or arithmetic mean of a baseline set of single-stimulation MEPs, respectively. Importantly, if $X$ is the MEP evoked by a paired stimulation, then $\delta$ and $\varrho$ effectively quantify the degree of intracortical inhibition or facilitation induced.

### Data processing

TMS data were cleaned to preserve only two relevant features: the peak-to-peak EMG of each stimulation and the diagnostic group (HC or MDD) of the participant upon which the stimulation was elicited. Outliers were removed following the IQR criteria on MEP amplitudes. The Julia programming language was used to add the ISI of each stimulation (with 0 encoding single pulses) and to compute $\delta$ and $\varrho$ for each stimulation. On each TMS session, the baseline set of stimulations used to compute $\delta$ and $\varrho$ were the single stimulations within that session. Thus, after data processing, each data sample consisted of a MEP amplitude, an ISI, and values for $\delta$ and $\varrho$. The total number of samples in the resulting data was $N=2855$.

### Statistical analysis

To characterize the distribution of MEP amplitudes, values were first averaged within each subject to account for repeated measures. These subject-level means were then stratified by ISI and experimental group. For each ISI × group combination, we assessed whether the resulting distribution followed a Gamma distribution. To this end, we performed a Kolmogorov–Smirnov (KS) test against the null hypothesis that the data were drawn from a Gamma distribution fitted via maximum likelihood estimation. The Gamma distribution was selected based on exploratory data analysis, including histograms, violin and Q–Q plots.

To assess the relationship between the likelihood of MDD and the independent predictors MEP amplitude, $\delta$ and $\varrho$, we used logistic regression with a linear mixed-effects model. These predictors have substantial pairwise correlations, indicating that their shared variance might bias regression estimates if included simultaneously in the model. To address this multicollinearity, we used principal component analysis (PCA) on the standardized values of these variables. The principal component which captured the greatest amount of variance, henceforth termed $P{C}^{(1)}$, was retained as the sole predictor of diagnostic status.

After PCA, the $P{C}^{(1)}$ predictor was grouped by and averaged within subjects and ISIs. The resulting aggregated data had a total of 294 observations. The following model was fitted via maximum likelihood estimation on this aggregated data:

$${logit}(P({y}_{{ij}}={MDD}))={\beta }_{0}+{\beta }_{1}P{{C}_{{ij}}}^{(1)}+{b}_{0j}+{b}_{1j}{{P}_{{ij}}}^{(1)}$$

where $i$ ranges across samples, $j$ across levels, ${b}_{0j}$ is a random intercept for ISI level $j$ and ${b}_{1j}$ is a random slope for $P{{C}_{{ij}}}^{(1)}$ at ISI level $j$. Thus, the model allows for the effect of $P{{C}_{{ij}}}^{(1)}$ to vary by ISI level. The model was fitted using the R programming language.

### Machine learning models

A Gradient Boosting classifier with 250 estimators (learning rate of 0.1, max. depth of 8) was trained to predict diagnostic status using 10-fold stratified cross validation. The model was implemented in Python using the scikit-learn library. The model was first trained on raw MEP data and ISI, then on $\delta$ and $\varrho$ along with ISI, and then on the combination of these features, giving a total of three distinct models with equal parametrization but distinct feature sets. The models were trained and tested on identical data splits. Thus, we assessed a. if raw TMS measures of cortical excitability were sufficient to predict MDD status; b. if $\delta$ and $\varrho$ were sufficient to predict MDD status; and c. to what degree their combination outperformed the other models.

### Performance metrics

We evaluated the performance of the models on each fold using measures aside from standard accuracy (correct predictions/total predictions). Because our dataset was imbalanced, we calculated the balanced accuracy score (average recall per class) and Cohen’s K. Matthew’s correlation coefficient (MCC), which measures the correlation between observed and predicted binary classifications and is robust to imbalanced datasets, was also computed. F1 scores (harmonic mean of precision and recall) were also used. We performed receiver operating characteristics curve (ROC) analysis. The Area Under the ROC (AUC) summarizes the overall discriminative power, with higher AUC values indicating superior performance. Due to class imbalances, we also computed the area under the precision-recall curve (PRC). All these metrics were then averaged across folds to obtain a single, mean performance metric for each model.

### Feature importance metrics

In the model trained over all features, we evaluated the contribution of MEP, $\delta$ and $\varrho$ to the model’s predictions using Shapley Additive Explanations (SHAP). For each fold, after training, SHAP values were computed for the held-out test subset using the SHAP Python library. These values allow us to identify which features consistently influence the model’s decisions across folds and are critical to ensure its interpretability. SHAP values from all test folds were aggregated to obtain an overall measure of feature importance.

Since $\delta$ and $\varrho$ are collinear and multicollinearity affects the interpretability of SHAP values [41], the SHAP values of $\delta$ and $\varrho$ were summed to aggregate their contributions and treat them as a single, combined feature. This provides a more stable measure of their collective impact on the model’s output.

The Gini feature importance (GFI) was computed for each model. This method estimates the importance of each feature by the degree to which it reduces the loss function across estimators. As SHAP values, GFI coefficients were computed for each cross-validation fold and averaged across folds to obtain a global measure.

### Model comparison

Paired t-tests were conducted to evaluate whether the distributions of each performance metric differed significantly across models. Specifically, metrics from the model trained on MEPs and ISI were compared to those from the model trained on ISI, $\delta$ and $\varrho$. Subsequently, the metrics from the latter model were compared to those of the model trained on all the features combined.

## Results

### KS tests and mixed-effects linear model

In both groups, and across all ISIs, the Kolmogorov-Smirnov tests indicated no significant deviation from the Gamma distribution (all p-values were greater than 0.15).

The mixed-effects linear model converged with an Akaike information criterion of 400.3 and a Bayesian information criterion of 418.7. Examination of the random effects revealed near-zero variance estimates: the random intercept variance was exactly zero, and the variance of the random slope for $P{C}^{(1)}$ was negligible (1.005 × 10^−20^), suggesting no meaningful variability in either the baseline log-odds or the effect of $P{C}^{(1)}$ across ISIs.

Fixed effects estimates indicated that PC^(1)^ was a statistically significant predictor of group membership. The estimated intercept was 0.442 (SE = 0.123, z = 3.60, p < 0.001), and the effect of $P{C}^{(1)}$ was 0.274 (SE = 0.109, z = 2.52, p = 0.012), suggesting that higher values of $P{C}^{(1)}$ were associated with increased odds of belonging to the MDD group.

### MDD predictions using machine learning

**Fig. 1:**
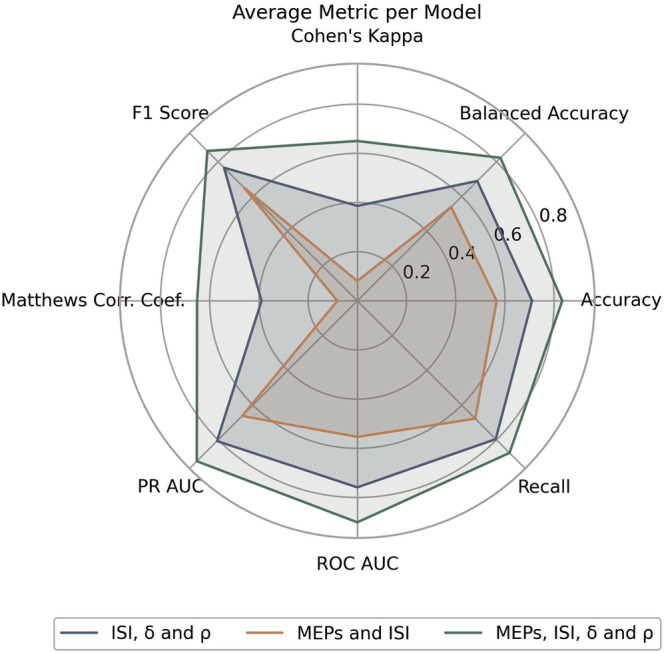
The spider plot summarizes performance of each model (distinguished by color) across eight evaluation metrics: Accuracy, Balanced Accuracy, Recall, ROC AUC, PR AUC, MCC, F1 Score, and Cohen’s Kappa. Each of the plotted metrics is the average across the 10 folds of its model. *t*-tests confirmed the differences between models were statistically significant (p-value < 0.001 for all metrics).

The performance metrics of the ML models are summarized in Table 1 and compared in Fig. 1. Particularly, in the model trained on MEP amplitude and ISI, the prediction of MDD status had an overall accuracy of 56.6% and a balanced accuracy of 53.9%. Cohen’s K was 0.08, indicative of virtually no agreement between the classifiers in the ensemble. F1 score was 0.651 while recall score was 0.678. The areas under the ROC and PR curves were 0.553 and 0.662, respectively.

**Table 1:** Model performance metrics.

Model’s features	Acc.	Balanced Acc.	Cohen’s *K*	MCC	F1	Recall	AUC	AUC-PR
MEP amplitude and ISI	0.566	0.539	0.080	0.081	0.651	0.678	0.553	0.662
$\delta ,\varrho$ and ISI	0.710	0.689	0.386	0.389	0.766	0.795	0.759	0.807
All of the above	0.833	0.823	0.650	0.651	0.862	0.875	0.901	0.923

In the model trained on ISI, $\delta$ and $\varrho$, overall accuracy was 71% and balanced accuracy was 68.9%. Cohen’s K was 0.386, indicating a fair agreement between the raters. F1 score was 0.766 while recall score was 0.795. The areas under the ROC and PR curves were 0.759 and 0.807 respectively.

**Fig. 2:**
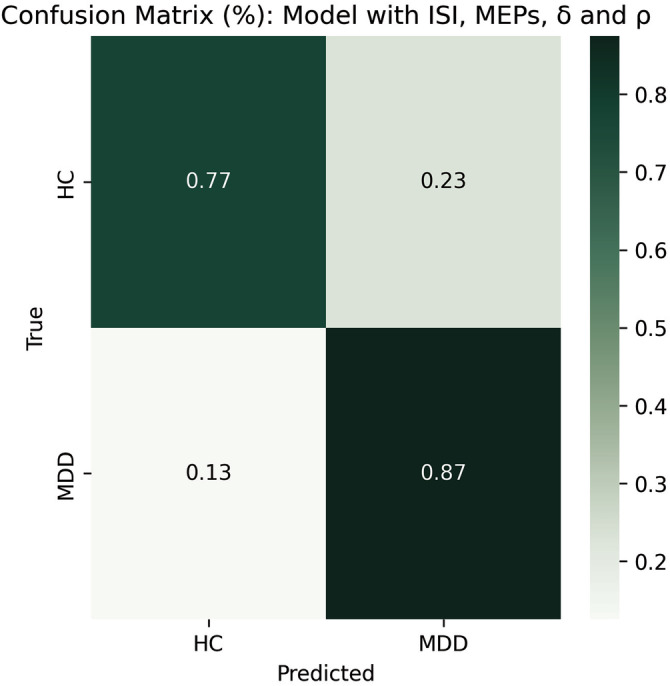
Normalized confusion matrix for the model trained on ISI, MEPs, *δ* and *ϱ.* The cells display the percentage of correct and incorrect classifications for each class (HC and MDD).

In the model trained on ISI, MEP amplitudes, $\delta$ and $\varrho$, accuracy was 83.3% and balanced accuracy 82.3%. Cohen’s K was 0.65, indicative of substantial agreement among raters. F1 and recall scores were 86.2 and 87.5, while the area under the ROC and PR curves were 0.901 and 0.923, respectively. In particular, the confusion matrix of this model (Fig. 2) revealed that predictions of MDD were correct 87% of times.

Paired t-tests comparing the distribution of each metric across models showed the performance of each model was significantly different from that of its predecessor. Specifically, the model with $\delta$ and $\varrho$ showed significantly superior performance metrics compared to the model trained on MEP amplitudes (p < 0.001 for each metric), and the model with all features combined also performed significantly better than the model that included $\delta$ and $\varrho$ without MEPs (p < 0.001 for each metric).

### Feature importance and SHAP value analysis

In the model which combined all features, Gini’s feature importance (mean decrease in impurity) revealed that $\delta$ and $\varrho$ were equally important (0.31 and 0.29 respectively), with MEP amplitude being slightly more important (0.36) (Table 2). The negligible remainder was attributed to ISI. SHAP value analysis consistently showed that high MEP amplitudes as well as high values of $\delta$ and $\varrho$ inclined the model towards a prediction of MDD. Conversely, low MEP amplitudes and low values of $\delta$ and $\varrho$ inclined the model towards the HC category.

**Table 2:** Feature importance (Gini) per model.

Model’s features	$\delta$	MEP amplitude	ISI	$\varrho$
MEP amplitude and ISI	NA	0.70	0.31	NA
$\delta ,\,\varrho$ and ISI	0.48	NA	0.06	0.46
All of the above	0.32	0.34	0.03	0.31

## Discussion

These results reveal that MEP amplitudes combined with our metrics $\delta$ and $\varrho$, which express the ratio of MEPs to some average baseline, effectively predict MDD in a simple ML model. We showed that even though $\delta$ and $\varrho$ are derived from MEPs, MEPs themselves were not sufficient to predict MDD. This suggests that $\delta$ and $\varrho$ effectively extract MDD-related information otherwise obscured in the raw data. Therefore, $\delta$ and $\varrho$ may potentially constitute two novel biomarkers of MDD.

The development of biomarkers of diagnostic status or treatment response in MDD is widely regarded as crucial for the prevention and personalized treatment of the condition [42–45]. However, the heterogeneous presentations of MDD make it difficult to define a single, or even a small set of functional biomarkers. MDD is associated with sleep abnormalities [46], endocrine and metabolic factors [47], imbalances in neurotransmitter levels [48], as well as social and relational conditions [49]. For this reason, the development of a diverse panel of distinct biomarkers that can index a wide variety of functional impairments is needed [36].

Impairments in neuroplasticity are a core feature of MDD [15, 48, 50]. Previous studies have shown that response to therapeutic TMS correlates with a reduction of interhemispheric excitability imbalances [44], and is predicted by the magnitude of induced corticomotor plasticity [45, 51]. Since biomarkers proposed to date, such as fMRI signal fluctuations [4–6], inflammatory cytokines [7, 8] or BDNF [9–11], are not directly associated with synaptic dysfunction, TMS-based biomarkers allow us to proxy a so far unrepresented aspect of MDD. In probing cortical excitability through TMS-evoked MEPs, $\delta$ and $\varrho$ may therefore fill an important gap in a potential biomarker panel.

**Fig. 3:**
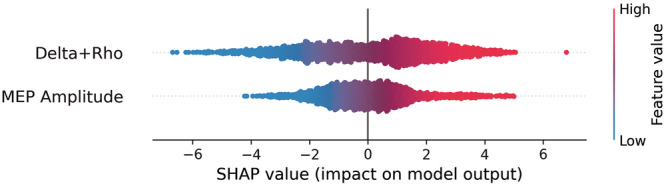
The SHAP summary plot illustrates the combined impact of *δ* and *ϱ* as well as the impact of MEP amplitude on the model’s output. Each dot represents a single observation. The x-axis denotes the SHAP value (magnitude and direction of influence), while the color gradient indicates the feature value (red = high, blue = low). Positive *x* values correspond to predictions of MDD, negative *x* values to predictions of healthy status. Both features demonstrate a positive relationship with MDD prediction.

High MEP amplitude, $\delta$ and $\varrho$ were predictive of MDD in the SHAP analysis (Fig. 3), mirroring the mixed-effects model where the principal component of these variables increased the odds of diagnosis. Interestingly, previous literature suggests that MDD is characterized by reduced GABAergic activity [16]. Since GABA is the main inhibitory neurotransmitter in the brain, this has led to several studies exploring hyper-excitability in MDD. Specifically, TMS studies report that depressed adults and adolescents show lower GABA_B_ receptor-mediated cortical inhibition relative to young adults [52], while some patient groups show increased intracortical facilitation [53]. A plausible interpretation of our SHAP values is that our model is using cortical hyperexcitability—driven by underlying GABAergic dysfunction and proxied by high MEP, $\delta$ and $\varrho$ values—to predict MDD. However, this is only one possible interpretation. It is important to note that GABA was not measured in this study, and our measures of corticospinal excitability are at best proxy measures of neural excitability. The precise role of GABA in MDD is complex, with some studies reporting conflicting findings, such as reduced facilitation [26], and variations across different subtypes of the condition [54].

In allowing for the training of machine learning models, $\delta$ and $\varrho$ potentially belong to an emerging class of ML-based biomarkers [43]. Though some of these biomarkers are successful, they typically fail to be interpretable, use tremendous amounts of high-dimensional and complex data, and fail to perform on imbalanced data [55]. Low-dimensional models trained over simple metrics are often preferable to complex and high-dimensional ones [56]. The models used in this research satisfy this principle of minimal complexity: only four features (MEP amplitude, ISI, $\delta$ and $\varrho$) were sufficient to consistently predict MDD using a relatively small and imbalanced sample. Furthermore, since TMS is already established as a therapeutic tool in MDD, they may be seamlessly integrated into clinical practice.

ML has been used with TMS in the past, but thus far, only in predicting therapeutic response to repeated TMS (rTMS) [57–59]. Moreover, these models did not use TMS data, but electroencephalography data collected before and after rTMS. It is plausible that TMS data itself has not so far been considered in ML models precisely due to the methodological limitations which $\delta$ and $\varrho$ were designed to overcome, including loss of data resolution and a sacrifice of within subject, between-subject, and across session variability.

Despite their predictive success, $\delta$ and $\varrho$ must be considered in light of certain limitations. Firstly, though significant work exists in the biophysical modelling of TMS-evoked MEPs [19], the numerous neurobiological factors modulating these responses remain poorly understood [41]. Therefore, $\delta$ and $\varrho$ are by necessity agnostic about the underlying physiology of corticospinal excitability. Though this agnosticism has advantages, it also introduces the risk of oversimplification. $\delta$ and $\varrho$ are based on TMS-evoked MEPs, which measure corticospinal excitability and proxy cortical excitability. $\delta$ and $\varrho$ collapse these layers of inference into single, unidimensional metrics.

Secondly, in order to be a good biomarker, the candidate must not only show clinical validity but also specificity. The specificity of $\delta$ and $\varrho$ with regard to MDD has yet to be determined. Future research should establish (a) whether they predict other psychiatric disorders characterized by impairments in cortical excitability and (b) whether they fail to discriminate conditions that are not associated with such impairments. Notwithstanding, even if $\delta$ and $\varrho$ fail to show specificity, they may still constitute valid, transdiagnostic biomarkers of synaptic dysfunction. Since synaptic dysfunction is posited to be a common feature of many psychiatric disorders, $\delta$ and $\varrho$ could still have value in facilitating dimensional, rather than categorical, diagnoses [60].

Lastly, MDD is known to be heterogeneous and characterized by several, potentially overlapping subtypes [61]. Future research should therefore investigate whether $\delta$ and $\varrho$ could not only predict MDD but also distinguish between some of its subtypes.

We recognize the existence of practical challenges in deploying these biomarkers as a diagnostic tool. Currently, TMS is typically administered as a treatment after a diagnosis of MDD has been made. Since TMS is expensive and requires trained personnel, one could argue that traditional standard structured interviews are still the preferable diagnostic tool.

However, one must consider $\delta$ and $\varrho$ not as standalone biomarkers which aim to replace structured interviews, but rather as potential components of a wider panel of biomarkers. The development of heterogeneous biomarker panels is considered to be crucial in multifactorial conditions such as MDD [36]. Viewed in this context, $\delta$ and $\varrho$ address a crucial shortfall: they are based on a potential physiological symptom thus far unconsidered in biomarker research for MDD.

Finally, although $\delta$ and $\varrho$ achieved promising discriminative performance, the small sample size used limits the generalizability of these findings. The risk of overfitting was mitigated by our use of 10-fold cross-validation, and since our performance metrics were averages across the 10 independent tests, they provide a more reliable estimate than metrics emerging from a single train/test split. However, we did not use out-of-sample validation sets, and future research is needed to fully determine how well the model generalizes to different samples.

In summary, this study demonstrated that $\delta$ and $\varrho$ allowed ML models to effectively predict MDD status from TMS-derived data with an accuracy of 83% and a balanced accuracy of 82%. This predictive success was achieved with a low-dimensional model trained on a small dataset. Therefore, $\delta$ and $\varrho$ may not only improve the ability to predict MDD but may also pave the way for new research exploring the use of TMS data itself in the prediction of diagnostic status or treatment response.

### Citation diversity statement

The authors have attested that they made efforts to be mindful of diversity in selecting the citations used in this article.

## Supplementary information

SUPPLEMENTARY MATERIAL

## Data Availability

The data that support the findings of this study are available from the corresponding author, JG, upon reasonable request.
