# Supplementary material for: Novel TMS-derived metrics enable machine learning classification of major depressive disorder
Source: NPP Digit Psychiatry Neurosci. 2026 Jan 12;4:1. doi: 10.1038/s44277-025-00053-w (PMC12796298; doi:10.1038/s44277-025-00053-w)
Supplement: Supplementary file 1 — SUPPLEMENTARY MATERIAL [file 44277_2025_53_MOESM1_ESM.pdf]

In the original article, we let $X \sim \Gamma(\alpha_X, \theta_X)$ denote the peak-to-peak amplitude of the MEP evoked by a (single or paired) TMS, and $\mathbf{T} := (T_1, \ldots, T_m)$, with $T_i \sim \Gamma(\alpha_T, \theta_T)$ $(1 \leq i \leq m)$ the peak-to-peak amplitude of the MEP evoked by m single, distinct stimulations. We allowed that $X = T_i$ for some i, in which case $\alpha_X = \alpha_T, \theta_X = \theta_T$. Then we defined

$$\varrho := \frac{Xm}{\sum_{i=1}^{m} T_i}, \qquad \delta := \frac{X}{m} \sum_{i=1}^{m} \frac{1}{T_i} \tag{1}$$

Here we present some mathematical properties of δ and ϱ considered as random variables. We henceforth assume that the random variables in $\mathbf{T}$ are independent and, in the case where $X \notin \mathbf{T}$, that X is independent from them as well. The case where $X \in \mathbf{T}$ and the case where $X \notin \mathbf{T}$ need to be treated separately.

Case $X \in \mathbf{T}$

Assume $X = T_k$ for some k $(1 \leq k \leq m)$. The distribution of δ is intractable, but since the ratio of a Γ-distributed variable to the sum of independent, identically distributed Γ variables is a β-distributed variable, we have

$$\frac{\varrho}{m} = T_k \left(T_k + \sum_{\substack{i=1,\\ i \neq k}}^{m} T_i \right)^{-1} \sim \beta(\alpha_T, (m-1)\alpha_T) \tag{2}$$

This entails that ϱ follows a scaled beta distribution:

$$\varrho \sim m \cdot \beta(\alpha_T, (m-1)\alpha_T) \tag{3}$$

This distribution has known mean and variance, and in particular

$$\mathbb{E}[\varrho] = \frac{m\alpha_T}{\alpha_T + (m-1)\alpha_T)} = 1 \tag{4}$$

This is reasonable, since we do not expect T_k to deviate from the baseline degree of excitability set by $\mathbf{T}$ in the case $T_k \in \mathbf{T}$.

Case $X \notin \mathbf{T}$

If $X \notin \mathbf{T}$, then the distribution of δ is once again intractable. Yet, since the ratio of two independent Gamma variables follows a scaled β-prime distribution, we have:

$$\frac{\theta_T \varrho}{m\theta_X} \sim \beta'(\alpha_X, m\alpha_T) \tag{5}$$

as long as $\alpha_T > 0, \alpha_X > 0$. The expected degree of excitability can be expressed as a ratio of means and as a mean of ratios:

$$\psi := \frac{\mathbb{E}[X]}{\mathbb{E}[T_i]} = \frac{\alpha_X \theta_X}{\alpha_T \theta_T}, \qquad \phi := \mathbb{E}\left[\frac{X}{T_i}\right] = \frac{\alpha_X \theta_X}{\theta_T(\alpha_T - 1)} \tag{6}$$

By the linearity of the expectation,

$$\lim_{m \to \infty} \mathbb{E}\left[\varrho\right] = \lim_{m \to \infty} \frac{m \alpha_X \theta_X}{\theta_T(m \alpha_T - 1)} = \psi, \qquad \mathbb{E}\left[\delta\right] = \frac{\alpha_X \theta_X}{\theta_T(\alpha_T - 1)} = \phi. \tag{7}$$

where the last equation only holds if $\alpha_T > 1$. Thus, under appropriate assumptions, the sample mean of ϱ converges to ψ as $m \to \infty$, and that of δ is an unbiased estimator of ϕ.
